# Systemic Screening for 22q11.2 Copy Number Variations in Hungarian Pediatric and Adult Patients With Congenital Heart Diseases Identified Rare Pathogenic Patterns in the Region

**DOI:** 10.3389/fgene.2021.635480

**Published:** 2021-04-29

**Authors:** Gloria Kafui Esi Zodanu, Mónika Oszlánczi, Kálmán Havasi, Anita Kalapos, Gergely Rácz, Márta Katona, Anikó Ujfalusi, Orsolya Nagy, Márta Széll, Dóra Nagy

**Affiliations:** ^1^Department of Medical Genetics, Faculty of Medicine, University of Szeged, Szeged, Hungary; ^2^Second Department of Internal Medicine and Cardiology Centre, Faculty of Medicine, University of Szeged, Szeged, Hungary; ^3^Department of Pediatrics, Faculty of Medicine, University of Szeged, Szeged, Hungary; ^4^Division of Clinical Genetics, Department of Laboratory Medicine, Faculty of Medicine, University of Debrecen, Debrecen, Hungary

**Keywords:** 22q11.2 deletion syndrome, *TBX1* gene, multiplex ligation-dependent probe amplification, copy number variations, droplet digital PCR, syndromic and non-syndromic congenital heart defects, chromosomal microarray analysis

## Abstract

Congenital heart defects (CHD) are the most common developmental abnormalities, affecting approximately 0.9% of livebirths. Genetic factors, including copy number variations (CNVs), play an important role in their development. The most common CNVs are found on chromosome 22q11.2. The genomic instability of this region, caused by the eight low copy repeats (LCR A-H), may result in several recurrent and/or rare microdeletions and duplications, including the most common, ∼3 Mb large LCR A-D deletion (classical 22q.11.2 deletion syndrome). We aimed to screen 22q11.2 CNVs in a large Hungarian pediatric and adult CHD cohort, regardless of the type of their CHDs. All the enrolled participants were cardiologically diagnosed with non-syndromic CHDs. A combination of multiplex ligation-dependent probe amplification (MLPA), chromosomal microarray analysis and droplet digital PCR methods were used to comprehensively assess the detected 22q11.2 CNVs in 212 CHD-patients. Additionally, capillary sequencing was performed to detect variants in the *TBX1* gene, a cardinal gene located in 22q11.2. Pathogenic CNVs were detected in 5.2% (11/212), VUS in 0.9% and benign CNVs in 1.8% of the overall CHD cohort. In patients with tetralogy of Fallot the rate of pathogenic CNVs was 17% (5/30). Fifty-four percent of all CNVs were typical proximal deletions (LCR A-D). However, nested (LCR A-B) and central deletions (LCR C-D), proximal (LCR A-D) and distal duplications (LCR D-E, LCR D-H, LCR E-H, LCR F-H) and rare combinations of deletions and duplications were also identified. Segregation analysis detected familial occurrence in 18% (2/11) of the pathogenic variants. Based on in-depth clinical information, a detailed phenotype–genotype comparison was performed. No pathogenic variant was identified in the *TBX1* gene. Our findings confirmed the previously described large phenotypic diversity in the 22q11.2 CNVs. MLPA proved to be a highly efficient genetic screening method for our CHD-cohort. Our results highlight the necessity for large-scale genetic screening of CHD-patients and the importance of early genetic diagnosis in their clinical management.

## Introduction

Congenital heart defects (CHDs) are the most common congenital developmental defects and affect approximately 0.9% of livebirths (van der Linde et al., 2011). Thirty to forty percent of CHDs are syndrome-associated and are caused by copy number variants (CNVs) or a mutation in a single gene. The most common human CNVs affect chromosomal region 22q11.2 (Fahed et al., 2013; Digilio and Marino, 2016). Proximal microdeletions of 1.5–3 Mb in this chromosomal region typically include the sequence between low copy repeat regions A and D (LCR A-D, LCR A-B) and may lead to the classical phenotype of 22q11.2 deletion syndrome, also known as DiGeorge syndrome. Central (LCR B-D, LCR C-D) or distal deletions (LCR C-H) may cause other, variable phenotypes (Burnside, 2015; Kruszka et al., 2017). Duplications have also been identified in this chromosomal region and are associated with even more significant phenotypic variability than deletions (Wentzel et al., 2008).

DiGeorge syndrome (also known as velocardiofacial syndrome and conotruncal anomaly face syndrome) is mostly characterized by CHD, thymus hypoplasia, immunodeficiency and skeletal, gastrointestinal and urogenital defects as well as by developmental delay, learning difficulties, susceptibility to neuropsychiatric disorders and, in some cases, by mild to moderate intellectual disability. 22q11.2 CNVs have reduced penetrance and incomplete expression and may be detected in asymptomatic or mildly affected individuals; approximately 7–10% of the cases are familial (McDonald-McGinn et al., 1993-2020; Campbell et al., 2018).

The *T-box transcription factor 1 (TBX1)* gene is located within the proximal 22q11.2 region, encodes a transcription factor that plays an important role in early embryonic development and is hypothesized to contribute to 22q11.2 deletion phenotype as well as to non-syndromic CHDs. (Griffin et al., 2010; Heike et al., 2010; Guo et al., 2011).

Clinical diagnosis may be challenging and significantly delayed due to the large phenotypic spectrum resulting from 22q11.2 CNVs (from asymptomatic appearance to multiple defects) (van Engelen et al., 2010). Previous studies have drawn attention to the importance of routine screening for 22q11.2 CNVs in patients with congenital heart defects, especially with conotruncal anomalies (Wozniak et al., 2010; Huber et al., 2014; Goldmuntz, 2020).

Based on the low number of patients referred for 22q11.2 CNV analysis at our genetic department over the last decade, we hypothesized that some patients with 22q11.2 CNVs—especially in the adult population—may have remained undiagnosed. The aim of our study was therefore to test for 22q11.2 CNVs and *TBX1* gene variants for the pediatric and adult patients of the Southern-Hungarian CHD Registry, cardiologically diagnosed with non-syndromic CHDs, and to carry out genotype–phenotype comparison in positive cases based on in-depth clinical data.

## Materials and Methods

Overall, 212 unrelated patients (110 females, 112 males; mean age: 26.9 years; age range: 2 weeks to 74 years) previously cardiologically diagnosed with non-syndromic congenital heart defects were enrolled in the study at the University of Szeged between 2016 and 2019. The distribution of the patients with the different CHD types are presented in Table 1.

**TABLE 1:** The distribution of different types of congenital heart defects among patients (*N* = 212).

Type of congenital heart defect	Number of patients (%)
**Ventricular septal defect**	**36 (16.9%)**
VSD alone	25
VSD + ASD + PDA	5
VSD + ASD	2
VSD + PDA	2
VSD + PS	2
**Atrial septal defect**	**35 (16.5%)**
ASD alone	27
ASD + PDA	3
ASD + VSD	3
ASD + PS	2
**Congenital aorta stenosis**	**31 (14.6%)**
AoS alone	17
AoS + bicuspid aortic valve	13
AoS + ASD	1
**Fallot IV**	**30 (14.1%)**
**TGA**	**21 (9.9%)**
**Bicuspid aortic valve**	**19 (8.9%)**
**Coarctation of the aorta**	**17 (8%)**
CoA alone	10
CoA + bicuspid aortic valve	3
CoA + VSD + PDA	4
Atrioventricular septal defect	5 (2.4%)
Anomalous pulmonary venous drainage	4 (2%)
TAPVD alone	2
TAPVD + PA + VSD	1
PPAVR	1
Pulmonary stenosis (congenital)	4 (2%)
Univentricular heart	3 (1.4%)
Hypoplastic left heart syndrome	2 (0.9%)
Pulmonary atresia	2 (0.9%)
Truncus arteriosus communis	1 (0.5%)
Double outlet right ventricle	1 (0.5%)
Ebstein anomaly	1 (0.5%)

**AoS, congenital aorta stenosis; ASD, atrial septal defect; CoA, coarctation of the aorta; Fallot IV, Tetralogy of Fallot; PA, pulmonary atresia; PDA, patent ductus arteriosus; PPAVR, partial pulmonary anomalous venous return; PS, pulmonary stenosis; TGA, transposition of the great arteries; TAPVD, total anomalous pulmonary venous drainage; VSD, ventricular septal defect. The most frequent CHD groups in the cohort are indicated in bold.**

The DNA of 211 Hungarian individuals with no CHDs (confirmed with cardiological examination), and with no family history of CHD (144 females, 67 males, mean age: 37 years, age range: 8–73 years) was used as controls for the comparative analyses.

In positive cases, genetic testing was offered to all first-degree family members.

All investigations were performed according to the Helsinki Declaration 2008 and approved by the National Medical Research Council (No CHD-01/2016—IF-6299-8/2016) and the Local Ethical Committee of the University of Szeged (No 105/2016-SZTE). Participants/legal guardians/parents gave their informed consent to the study.

### Sample Preparation and Multiplex Ligation-Dependent Probe Amplification (MLPA)

DNA was extracted from peripheral blood with the QIAamp DNA Blood Mini Kit (QIAGEN, Gödöllõ, Hungary).

To detect CNVs in the 22q11.2 locus, all patient samples were processed using the P250-B2 DiGeorge SALSA MLPA Probemix (IVD, MRC-Holland, Amsterdam) according to the manufacturer’s instructions. The MLPA probe mix contained 48 probes, 29 of which are located in the 22q11.2 region (24 in the LCR A to H region and 5 in the Cat-Eye syndrome region) and 19 in regions 4q35, 8p23, and 9q34 (Kleefstra syndrome), 10p14 (DiGeorge syndrome 2) and 17p13 and 22q13 (Phelan-McDermid syndrome), deletions in the latter may result in phenotypical similarity to DGS. Amplicon fragment length analysis was performed on an ABI 3500 Genetic Analyzer (Thermo Fisher Scientific, Waltham, MA) and analyzed by Coffalyser.net software (MRC-Holland, Amsterdam).

### Validation of Positive Cases: FISH, Chromosomal Microarray Analysis, ddPCR

MLPA was repeated for all samples in which CNVs were found. Deletions and duplications were confirmed with an independent method, including FISH (Vysis DiGeorge Region LSI N25 SO/ARSA SGn Probes, Abbott Molecular Inc., Des Plaines, IL, United States, and SureFISH 22q11.21 CRKL, Agilent Technologies, Cedar Creek, TX, United States), a supplementary MLPA kit (P372-SALSA MLPA Microdeletions 6, MRC-Holland, Amsterdam, Netherlands) or chromosomal microarray analysis (CMA, Affymetrix, CytoScan 750 K, Thermo Fisher Scientific, Waltham, MA, United States). CMA was performed as described by Nagy et al. (2019). In cases where one probe was deleted, the probe region was sequenced with bidirectional capillary sequencing to exclude MLPA-interfering SNPs in the sample DNA. These validation methods confirmed all positive MLPA results (i.e., no false positives).

A droplet digital PCR (ddPCR) method was designed for the confirmation of recurrent single-probe CNVs in the *TOP3B* gene from CHD patient samples. This method was also used to determine the frequency of *TOP3B* CNVs in the control cohort as well. The analysis was performed on the QX100 Droplet Digital PCR system (Bio-Rad Laboratories, Hercules, CA, United States), according to the manufacturer’s instruction. Primers and probes were designed for *TOP3B* exon 7 and for the *PRDM15* gene as reference region on chromosome 21 (Supplementary Material). *TOP3B* CNVs found in the controls with ddPCR were confirmed with MLPA.

### Sequencing of the *TBX1* Gene

Bidirectional capillary sequencing of *TBX1* coding regions was performed for all patient samples with an ABI 3500 Genetic Analyzer. The primers used are listed in Supplementary Material. The non-synonymous variants (all located in exon 9 of *TBX1* gene) were tested in the control cohort as well.

### CNV and Variant Interpretation

Identified CNVs and single nucleotide variants (SNVs) were classified according to the standards and guidelines of the American College of Medical Genetics (Richards et al., 2015; Riggs et al., 2020). The following websites and databases were used for CNV interpretation: Database of Chromosomal Imbalance, Phenotype of Humans using Ensemble Resources (DECIPHER, Firth et al., 2009), Database of Genomic Variation (DGV, MacDonald et al., 2013), PubMed and GeneReviews (McDonald-McGinn et al., 1993-2020). For SNV interpretation, VarSome (Kopanos et al., 2011), ClinVar (Landrum et al., 2020), and Genome Aggregation Database (GnomAD, Karczewski et al., 2020) databases were used.

### Statistical Analysis

GraphPad Prism (GraphPad Software, San Diego, California, United States), version 4.00 for Windows, was used for statistical analysis. The frequency of *TOP3B* CNVs and *TBX1* variants in the patient cohort was compared with the frequency in the control cohort and also with the frequency in the global dataset of GnomAD using the Fisher exact test and χ^2^-test. *P* < 0.05 was considered to be statistically significant.

## Results

### Distribution of CHD Types in Patients

In the CHD cohort, the four most common CHD types were ventricular septal defect (VSD), atrial septal defect (ASD), congenital aorta stenosis (AoS) and tetralogy of Fallot (TOF) (Table 1). In 81% of the patients, only one cardiac entity was diagnosed; whereas, in 19% of the cases, two or more CHDs occurred together. The distribution of the different CHDs among the South-Hungarian Registry patients corresponded well with the frequency described in the literature (van der Linde et al., 2011).

### Distribution of Positive MLPA Results and Classification of the Detected CNVs

**FIGURE 1:**
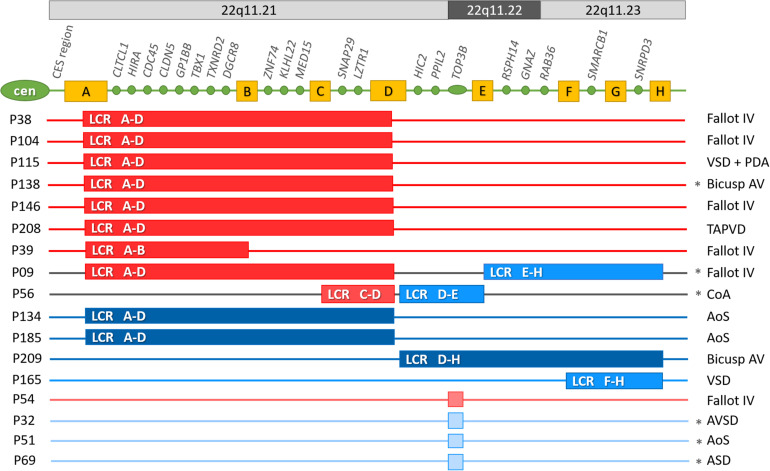
Pathogenic variants, variants of uncertain significance and benign 22q11.2 copy number variations in the CHD cohort. Cen, centromere; CES, Cat eye syndrome region; A-H yellow boxes: low copy repeat regions in locus 22q11.2. Genes indicated between the LCR regions, are the ones that have corresponding probes in the P250-B2 DiGeorge SALSA MLPA Probemix; LCR: low copy repeat region. Dark red: pathogenic microdeletion; Dark blue: pathogenic microduplication; Middle red: microdeletion of uncertain significance; Middle blue: microduplication of uncertain significance; Light red: benign microdeletion; Light blue: benign microduplication. LCR A-D: the typical ∼2.5–3 Mb microdeletion in region 22q11.21; LCR A-B: ∼1.5 Mb proximal microdeletion in region 22q11.21; LCR C-D: ∼0.5 Mb central microdeletion in region 22q11.21; LCR D-E: ∼1.2 Mb central microduplication in region 22q11.21q11.22; LCR D-H: ∼3.1–3.5 Mb central-distal microduplication in region 22q11.21q23; LCR E-H: ∼1.55–2 Mb distal microduplication in region 22q11.22q11.23; LCR F-H: ∼1–1.2 Mb distal microduplication in region 22q11.23; Asterix denotes familial CNVs. AoS, congenital aorta stenosis; AVSD, atrioventricular septal defect; bicuspid AV: bicuspid aortic valve; CoA, coarctation of the aorta; Fallot IV, tetralogy of Fallot; PDA, patent ductus arteriosus; TAPVD, total anomalous pulmonary venous drainage; VSD, ventricular septal defect.

Overall, 17 cases of 212 patients (8%) diagnosed prior with non-syndromic CHD were yielded positive MLPA result, and after evaluation 11 of these copy number changes (5.2%) were interpreted as pathogenic variant, two as variant of unknown significance (VUS, 0.9%) and four as benign (1.8%) (Figure 1 and Supplementary Material). The most frequent CNVs of the positive MLPA results were microdeletions (8/17); however, microduplications (7/17) and a combination of deletions and duplications (2/17) were also observed.

Among pathogenic CNVs 7 microdeletions, 2 duplications and 1 combination of a deletion and a duplication was detected, while among the VUS one duplication and one combined CNV and among the benign variants one deletion and three duplications.

Pathogenic results were observed most frequently in the TOF group: in 17% of all TOF patients, followed by the group of bicuspid aortic valve with 10% (Figure 1 and Table 2).

**TABLE 2:** The distribution of the pathogenic and VUS 22q11 CNVs in the different CHD groups.

Type of CHD	Number of pathogenic CNVs or VUS/Number of patients with CHD (%)	Del	Dupl	Del + Dupl
**Fallot IV**	**5 path/30 (17%)**	**4**	0	**1**
Bicuspid aortic valve	2 path/19 (10%)	**1**	**1**	0
AoS	2 path/31 (6.5%)	0	**2**	0
CoA	1 VUS/17 (6%)	0	0	**1**
VSD	1 path/36 (2.7%)	**1**		0
	1 VUS/36 (2.7%)		**1**	
TAPVD + VSD + PA	1 path/4 (25%)*	**1**	0	0
**Total**	**11 path/212 (5.2%)**	**7**	**3**	**1**
	**2 VUS/212 (0.9%)**	**0**	**1**	**1**

VUS, variant of uncertain significance; CHD, congenital heart defect; CoA, coarctation of the aorta; AoS, congenital aorta stenosis Fallot IV, tetralogy of Fallot; TAPVD, total anomalous pulmonary venous drainage; VSD, ventricular septal defect; PA, pulmonary atresia; DEL, 22q11 microdeletion; DUPL, 22q11 microduplication. *This proportion is biased, since this patient could be also classified in VSD or PA group.

Based on the interpretation guidelines (Riggs et al., 2020), 11 CNVs were interpreted as pathogenic:

6 typical deletions of LCR A-D, one proximal nested deletion of LCR A-B, two duplications of LCR A-D, one combination of the proximal deletion of LCR A-D with a duplication of LCR E-H and one duplication of LCR D-H. Two further CNVs (one combination of a central deletion of LCR C-D with duplication of LCR D-E and one duplication of LCR F-H) were classified as VUS (Figure 1 and Table 3). Four CNVs (three 268 kbp duplications and one 278 kbp deletion) were detected in the *TOP3B* gene and resulted from one probe alteration in the MLPA reaction. These CNVs were confirmed with chromosomal microarray analysis (Supplementary Material). Considering the relatively high proportion of *TOP3B* CNVs in our patient cohort (overall 4/212, 1.9%: deletion in 1/212, 0.5% and duplication in 3/212, 1.4%), we decided to perform an independent analysis with ddPCR to determine the frequency of *TOP3B* CNVs in the healthy controls. The *TOP3B* deletion was detected in one control sample (0.5%) and a duplication in four control samples (1.9%); i.e., CNVs were identified in 2.4% of the controls (5/211). The difference in the CNV frequency between patients and controls was not significant (*p* = 0.751). Thus, we ultimately classified *TOP3B* CNVs as rare benign variants, which are more frequent in the Hungarian population than in the global database (frequency in DECIPHER: 0.36%).

**TABLE 3:** Clinical features of probands and parents with pathogenic and VUS MLPA results.

Proband no./Age*	CNV Extension (size)	Classifi- cation**	Type of CHD	Extracardiac manifestations	DD/ID	Facial features	Classical pheno-type***	Hypo-calcemia	Miscellaneous	Familial inheritance or *de novo* mutation
P38/Adulthood	**DEL LCR A-D** in 22q11.21 (∼2.5-3 Mb)	Path	TOF	Recurrent bronchitis and otitis media, tonsillectomy, vesicoureteral reflux, renal cyst, velopharyngeal insufficiency, nasal speech, early teeth lost, small stature, kyphoscoliosis, block vertebrae, lower leg cramps, dyslexia, anxiety disorder, microcephaly, juvenile cataract, autoimmune hypothyroidism	DD/mild ID	Narrow face, micrognathia, low-set ears, narrow, small palpebral fissures, hypertelorism, hypoplastic alae nasi, pointed ear tips, thin lips	Yes	Yes	Obstipation, GOR, feeding difficulties in childhood, hypomagnesemia	*De novo*
P104/Adulthood	**DEL LCR A-D** in 22q11.21 (∼2.5–3 Mb)	Path	TOF	Arrythmia (radio frequent ablation), recurrent otitis media in childhood (Grommet tubes, adenotonsillectomy), inguinal hernia, scoliosis, mild kyphosis, thorax asymmetry, narrow shoulders, recurrent urinary infections, nephrolithiasis, nasal speech, learning difficulties.	DD/low normal IQ	Narrow, long face, narrow palpebral fissures, deep-set eyes, marked hypertelorism, large ear lobes, thin small lip, hypoplastic alae nasi, malar flattening	Yes	Yes	Lumbago, pulmonary embolism. Deceased postoperative in nosocomial infection before the genetic diagnosis	*De novo*
P115/Adulthood	**DEL LCR A-D** in 22q11.21 (∼2.5–3 Mb)	Path	VSD, PDA	Right main bronchus stenosis, congenital lacrimal duct stenosis, many recurrent upper and lower airway infections until puberty, severe scoliosis, hernia diaphragm + severe GOR (fundoplication), inguinal hernia (operated), palatoschisis, velopharyngeal insufficiency, thorax and hip deformity, learning difficulties, nasal speech, episodic hand tremor and foot paresthesia, small stature, microcephaly	DD/low normal IQ	Narrow, long face low-set ears, narrow, small palpebral fissures, hypertelorism, hypoplastic alae nasi, pointed ear tips, thin small lips, malar flattening, mild facial asymmetry	Yes	−	Nasogastric tube feeding in infancy	*De novo*
P138/Childhood	**DEL LCR A-D** in 22q11.21 (∼2.5–3 Mb)	Path	Bicuspid aortic valve	Recurrent lower and upper airway infections and otitis media, nasal speech, hypermetropy, astigmia, no developmental delay, normal kindergarten, and preschool	No/No	Long face, small mouth, straight nose, narrow eyelids, hypertelorism, pointed ear tip, fleshy ear lobes	Yes	Yes	Severe obesity (due to diet failure), secondary hypertonia	Maternally inherited: mother has similar outer appearance, umbilical hernia, vestibular neuronitis, impaired hearing, but no CHD
P146/Adulthood	**DEL LCR A-D** in 22q11.21 (∼2.5–3 Mb)	Path	TOF	Recurrent respiratory infection and otitis media, mastoiditis, cryptorchism (orchidopexy), severe scoliosis, thorax asymmetry (small right scapula), nasal speech, neuropsychiatric problems, learning difficulties, special school	Speech delay/mild ID	Narrow, long face, straight nose, low-set ears, narrow palpebral fissures	Yes	Yes	Brain MRI and abdominal ultrasound: normal	*De novo*
P208/Childhood	**DEL LCR A-D** in 22q11.21 (∼2.5–3 Mb)	Path	TPAVD, PA, VSD	Thymus aplasia, many respiratory infections in small childhood, prolonged Candidiasis, small stature	DD/mild ID	Narrow face, micrognathia, low-set ears, narrow, small palpebral fissures	Yes	Yes	Epidermal skin problems	*De novo*
P39/Adulthood	**DEL LCR A-B** in 22q11.21 (∼2.5–3 Mb)	Path	TOF	Recurrent respiratory infections, learning difficulties, special school, neuropsychiatric problems	DD/low normal IQ	Narrow face, low-set ears, narrow, small palpebral fissures	Yes	−	–	ND
P09/Childhood	**DEL LCR A-D** in 22q11.21 (∼2.5–3 Mb) + **DUPL LCR E-H** 22q11.22q11.23 (∼1.55–2 Mb)	Path + VUS	TOF	Congenital laryngeal stenosis, thymus aplasia, respiratory infections (postoperative as well), pes calcaneovalgus	?/?	Small mouth, pointed ear tips, hypoplastic alae nasi, low-set ears	Yes	No	Small for gestational age, transient nasogastric tube feeding	Maternally inherited. mother: TOF, severe scoliosis, nasal speech, classical DGS phenotype, low normal IQ, anxiety disorder
P56/Adulthood	**DEL LCR C-D** in 22q11.21 (∼0.5 Mb) + **DUPL LCR D-E** 22q11.21q11.22 (∼1.2 Mb)	VUS + VUS	CoA	Torticollis, scoliosis, nasal speech, socially withdrawn, studies in higher education	No/No	Facial asymmetry, pointed ear lobes, small philtrum, low-set ears, triangular chin, retrognathia	No	No	–	Maternally inherited: mother has bicuspid aortic valve, similar facial features without asymmetry and torticollis
P134/Adulthood	**DUPL LCR A-D** in 22q11.21 (∼2.5–3 Mb)	Path	AoS	None	No/No	No	No	No	Atopy, asthma	*De novo*
P185/Adulthood	**DUPL LCR A-D** in 22q11.21 (∼2.5–3 Mb)	Path	AoS	None	No/No	No	No	No	–	ND
P209/Adulthood	**DUPL LCR D-H** 22q11.21q11.23 (∼3.1–3.5 Mb)	Path	Bicuspid aortic valve	Horseshoe kidney, pyeloureteral stenosis, Ewing-sarcoma in childhood, frequent tonsillitis (tonsillectomy), primary amenorrhea, special school	Speech delay/mild ID	Hypertelorism, divergent strabismus, prominent long mandible, uvula elongata	No	No	Obesity. Twin sibling died of pulmonary atresia after birth	No CNV in father, mother not tested
P165/Adulthood	**DUPL LCR F-H** in 22q11.23 (∼1–1.2 Mb)	VUS	VSD	Myopia, bilateral inguinal hernia, truncal obesity, learning difficulties	No/No	Micrognathia	No	No	Preterm birth, normal catch-up development, bronchial asthma	*De novo*

CHD, congenital heart disease; CNV, copy number variation; LCR, low copy repeat region; TOF, Tetralogy of Fallot; AoS, congenital stenosis of the aorta; PA, pulmonal atresia; VSD, ventricular septal defect; TPAVD, Total anomalous pulmonary venous return; CHD, congenital heart disease; CoA, coarctation of the aorta; AVSD, atrioventricular septal defect. ND, not done; DD, developmental delay; ID, intellectual disability, GOR, gastro-esophageal reflux; Path, pathological; VUS, variant of uncertain significance.

*Denotes the life period when the genetic diagnosis was set up. **Denotes the classification of CNVs based on the joint consensus of ACMG and ClinGen Guidelines (Riggs et al., 2020). ***Classical phenotype of 22q11.2 deletion syndrome, previously described as DiGeorge syndrome.

### Familial Segregation

It was possible to perform segregation analysis for 14 of the 17 positive cases. Six cases proved to be familial (Figure 1), two of these were for patients with pathogenic CNVs, one for a patient with VUS and three for patients with benign *TOP3B* variants. In addition to the *TOP3B* microduplication, proband P32 also had a 201-bp microduplication on chromosome 17p13.3, which included the *YWHAE* gene (Supplementary Material). Both chromosome imbalances were inherited from an asymptomatic parent. Two asymptomatic siblings also carried the *YWHAE* microduplication but without the *TOP3B* CNV. Therefore, the *YWHAE* CNV was interpreted as a rare benign variant. In case of the other *TOP3B* microduplications (P51 and P69) one healthy parent carried also the variant. In the patient with the *TOP3B* deletion, segregation analysis was not performed, the proband had two healthy children. The individuals with *TOP3B* CNVs were excluded from the genotype–phenotype comparison based on these results, the high frequency of *TOP3B* CNV in controls and the fact that these patients displayed no other malformations or comorbidities in addition to CHD.

The segregation analysis detected familial occurrence for 18% (2/11) of the pathogenic CNVs and 50% (1/2) of VUS. In these three familial cases (P138, P09 and P56), the proband’s mother carried the same chromosome imbalance. The phenotypes of the mothers were the same severity (P138) or milder (P09, P56) than the probands’. The suspicion of an underlying 22q11.2 CNV prior to the genetic testing was not raised at any of the affected family members.

For proband P09 and for the proband’s mother, the typical ∼2.5–3 Mb 22q11.2 microdeletion was combined with a distal ∼1.5–2 Mb 22q11.2 microduplication. The segregation analysis of the family showed that the maternal grandmother and one sibling of the mother carried only the duplication with no cardiological symptom or developmental malformation, although they had been diagnosed with mild anxiety disorder and depression. The deletion occurred most probably *de novo* in the mother and was transferred to the child.

The mother of proband P56 had only bicuspid aortic valve (clinically diagnosed only after the genetic diagnosis) and similar facial features as the proband without torticollis or severe scoliosis.

The clinical features of patients and family members are shown in Table 3.

### Genotype-Phenotype Comparison

The probands’ age at the genetic diagnosis with pathogenic or VUS 22q11.2 CNVs ranged from 2 months to 52 years (median age: 21 years). Three patients out of 13 were diagnosed in childhood, one child in the first year of life. The two oldest patients and the affected family members were born before the molecular diagnostic era. No correlation could be observed between the severity of the phenotype and the age at the diagnosis.

The prevalence of common clinical features for different CNVs is comparable to previously reported prevalence data in the literature (Table 4). Patients presented with more marked phenotypic features for 22q11.2 microdeletions than with microduplications in the same region. In addition to the CHDs, the typical microdeletions of LCR A-D—with or without accompanying CNVs—resulted in the classical phenotype of 22q11.2 deletion syndrome. The co-occurring duplication in proband P09 and mother has not modified their phenotype significantly compared to other LCR A-D microdeletion phenotypes. In proband P56 and his mother with the combination of central deletion and distal duplication, the phenotype differed completely from that of 22q11.2 deletion syndrome (except the CHD) and the impact of the duplication could not be determined precisely (Table 3).

**TABLE 4:** The prevalence of the common clinical features in all probands and family members carrying pathogenic CNVs and VUS in the 22q11.2 region compared to the prevalence in the literature.

Symptoms	Classical LCR A-D deletion	All other deletions*	All duplications alone	All CNVs of this study	Prevalence in the literature**
CHD	8/9 (89%)	3/3 (100%)	4/4 (100%)	**15/16 (94%)**	64–74%
Facial dysmorphia Classical facial features in 22q11.2 deletion	9/9 (100%) 9/9 (100%)	3/3 (100%) 1/3 (33%)	2/4 (50%) 0/4	**14/16 (87.5%) 10/16 (62.5%)**	46–88% ND
Velopharyngeal insufficiency	7/9 (78%)	1/3 (33%)	0/4	**8/16 (50%)**	55–69%
Immunodeficiency, recurrent infections	8/9 (89%)	1/3 (33%)	1/4 (25%)	**10/16 (62.5%)**	50–77%
Skeletal anomalies	6/9 (67%)	1/3 (33%)	0/4	**7/16 (44%)**	15–50%
Other anomalies***	7/9 (78%)	0/3	2/4 (50%)	**9/16 (56%)**	67–81%
Developmental delay (motor ± speech) in childhood	6/9 (67%)	1/3 (33%)	1/4 (25%)	**8/16 (50%)**	70–90%
Intellectual disability	4/9 (44%)	0/3	1/4 (25%)	**5/16 (31%)**	28–31%
Learning difficulties	6/9 (67%)	1/3 (33%)	1/4 (25%)	**8/16 (50%)**	66–93%
Neuropsychiatric problems	3/9 (33%)	0/3	0/4	**3/16 (19%)**	60–73%
Hypocalcemia	5/9 (55.5%)	0/3	0/4	**5/16 (31%)**	17–60%

CHD, congenital heart defect; ID, intellectual disability.

*In this category LCR A-B nested deletion and LCR C-D deletion combined with LCR D-E duplication are included; ** Data for 22q11.2 deletions described by McDonald-McGinn et al. (1993-2020), Burnside (2015); Campbell et al. (2018), and Niarchou et al. (2019) are presented; *** Including clinically diagnosed thymus aplasia, respiratory, gastrointestinal and/or urogenital abnormalities; ND: not determined. Velopharyngeal insufficiency, nasal speech could not be assessed in the two youngest probands. Intellectual disability, learning difficulties could be only assessed in individuals in school age or older. Psychiatric disorders are taken into account only if they were clinically diagnosed.

Among patients with deletions, Fallot tetralogy was the most common CHD. Among patients with duplications, congenital aorta stenosis, coarctation of the aorta and bicuspid aortic valve were the most common CHD types (Table 2). These three entities may be considered on the spectrum for one disease.

CHDs were overrepresented in our 22q11.2 CNV patients and their affected family members compared to data in the literature (94% vs. 74%), which may be the result of the patient enrollment criteria. Neuropsychiatric disorders were underrepresented among our patients (19% vs. 60%). Other characteristics (facial features, velopharyngeal insufficiency, immunodeficiency, hypocalcemia, skeletal anomalies, developmental delay, and learning difficulties) had a distribution in our cohort similar to that described in the literature (Table 4). The presence of immunodeficiency was deduced from the recurrence of respiratory and ear infections occurring mostly in childhood. Based on regular laboratory check-ups, the average absolute lymphocyte count was in the lower normal range (2.13 G/L, normal range: 1.5–3.2 G/L); whereas the average relative lymphocyte count was below normal (23.8%, normal range: 27–34%). Flow-cytometry and serum immunoglobulin levels were not measured regularly. The immune status and infections of 22q11.2 CNV patients were not strictly controlled before the genetic diagnosis. Before genetic diagnosis, proband P104 died of a fulminant postoperative infection (Table 3).

Hypocalcemia (average serum calcium level: 1.84 mmol/l, normal range: 2.2–2.55 mmol/l) was often present in patients with the typical 22q11.2 microdeletions—with or without clinical symptoms. However, hypocalcemia was not considered relevant for therapy before genetic diagnosis. Severe hypomagnesemia was also detected in one 22q11.2 microdeletion patient. The thrombocyte count was in the low normal range with an average of 156 × 10^9^/l. Thyroid and parathyroid hormone levels and vitamin D levels were not measured in these patients before genetic diagnosis.

These laboratory abnormalities could not be consistently identified for patients with CNVs other than the typical microdeletion.

### Results of the *TBX1* Gene Sequencing

No apparently pathogenic variant was detected in the *TBX1* gene. For CHD patients, three missense variants were found in exon 9: c.1189A>A; p.Asn397His with a 21% minor allele frequency (MAF), c.1049G>A; p.Gly350Asp with 0.48% MAF and c.1341_1342insCCGCACGCGCAT; p.Ala450_His453dup with 0.24% MAF (Table 5). The frequency of the p.Asn397His variant was also 21% for the controls. The two less frequent variants were also detected in one of the proband’s healthy parents and are listed in the Hungarian or the global database with very low frequencies (Table 5). Of the 10 probands and mothers with proximal 22q11.2 microdeletions encompassing the *TBX1* gene, two (20%) carried the common p.Asn397His variant in hemizygous form (P09 and P138). Proband P09 exhibited a severe phenotype; whereas proband P138 presented only milder symptoms. The two rare variants were not detected in any of the microdeletion patients. Based on the allele frequencies, ACMG criteria and segregation analyses, all three variants were ultimately classified as benign.

**TABLE 5:** Minor allele frequencies of *TBX1* variants in the CHD cohort and control cohort compared to the allele frequency in the global database.

Variant	MAF in CHD	MAF in controls	*p*	MAF in GnomAD	*p*	*In silico* prediction*
c.1189A>A; p.Asn397His (rs72646967)	21%	21%	*0.809*	23.19%	*0.3583*	Benign
c.1049G>A; p.Gly350Asp (rs781731042)	0.48%	0.95%	*0.686*	0.0402%	*0.0138*	Benign
c.1341_1342insCCGCACGCGCAT; p.Ala450_His453dup (rs1341195668)	0.24%	0%	*0.498*	0.00325%	*0.0267*	VUS

*In TBX1 locus CHD cohort contained overall 418 alleles, control cohort 422 alleles.*

*MAF, minor allele frequency; CHD, congenital heart defect.*

GnomAD, Genome Aggregation Database (Karczewski et al., 2020). TBX1 transcript number: NM_080647. *In silico variant prediction was performed with VarSome, based on the ACMG criteria (Kopanos et al., 2011; Richards et al., 2015).

## Discussion

This was the first systemic, large-scale genetic screening study of Hungarian CHD patients. All patients with cardiologically verified CHDs were enrolled in the study without further selection. Although the enrolled patients were cardiologically diagnosed with non-syndromic CHDs prior to this study, 13 were found to be syndromic after the genetic screening.

We observed a higher median age (21 years) and a similar or wider age range (0.17–52 years) at the genetic diagnosis in our cohort as compared to previously described cohorts (median age: 17.3 years, range: 0.1–59.4 years in Canadian patients; median age: 2.9 years, range: 0–17.6 years in American patients) (Palmer et al., 2018). This difference may partly be explained by the fact, that the 22q11.2 duplication patients with more variable phenotypes were also included in the present study, whereas only 22q11.2 deletions were analyzed by Palmer and colleagues.

The frequency of CHDs was representative and corresponded to the frequency described in large epidemiological studies (van der Linde et al., 2011).

All types of CNVs in the 22q11.2 chromosomal region were present in 8% of the CHD cohort, while pathogenic CNVs in 5.2%, VUS in 0.9% and benign CNVs in 1.8%. Our patients presented pathogenic 22q11.2 CNVs more often compared to other CHD cohorts, such as 1.27% in Brazilian, 2.8% in Cameroonian and 2.9% in Chinese population (Huber et al., 2014; Wonkam et al., 2017; Li et al., 2019). However, this difference may be also explained by the fact that most of these studies focused on the detection of 22q11.2 deletion but not on duplications.

For tetralogy of Fallot, the proportion of pathogenic CNVs was significantly higher, 17% in our cohort, which is in agreement with the fact that 22q11.2 CNVs are common in conotruncal heart defects (McDonald-McGinn et al., 1993-2020). 22q11.2 deletion can be detected in approximately 20% of all conotruncal heart defects (Wozniak et al., 2010), within this category its prevalence can be as high as ∼50% in interrupted aortic arch type B, ∼35% in truncus arteriosus or 10–25% in tetralogy of Fallot (Goldmuntz, 2020). All these suggest an absolute indication for 22q11.2 CNV analysis in these CHD groups, especially when co-occurring with at least one extracardiac manifestation or dysmorphic traits (Wozniak et al., 2010).

Congenital bicuspid aortic valve is common (0.5–2%) and, without complication of stenosis, regurgitation or dissection, considered a largely benign congenital heart defect (Li et al., 2017). However, based on our results, it should not be ignored in genetic testing.

The most common (64%) pathogenic CNV among our patients was the typical microdeletion of the LCR A-D region on chromosome 22q11.2, which is in agreement with the literature (Du et al., 2020). The frequency of deletions decreased toward the LCR F-H region, which was reflected in our results as well, since nested and central deletions were rare, and distal deletions were not detected. Proximal and distal duplication as well as two combined CNVs were also identified. Although most patients with the typical LCR A-D deletion showed the majority of the characteristic features of 22q11.2 deletion syndrome (velopharyngeal insufficiency, skeletal malformation, gastrointestinal and nephrological anomalies, hypocalcemia, frequent infections due to immunodeficiency and common facial features), these symptoms were present less frequently (Table 4) with the non-typical deletions and the duplications, as expected (Burnside, 2015; Du et al., 2020). In addition to the presence of CHDs, no typical common characteristics could be found for these patients. This may be due to the low number of patients with single CNVs in our cohort or to the even wider phenotypic spectrum of these CNVs, e.g., in the case of 22q11.2 duplications (Yu et al., 2019). Therefore, several individuals with only mild symptoms or no detectable malformation or dysmorphia may remain undetected.

Hypocalcemia, hypomagnesemia, lymphocytopenia, thrombocytopenia, and abnormalities of the thyroid, parathyroid hormone or vitamin D levels may remain undiscovered in 22q11.2 deletion patients, especially without genetic diagnosis. However, these conditions may significantly contribute to co-morbidities, such as increased susceptibility to infections, bleeding diathesis and heightened prevalence of autoimmune disorders, and, thus, should be considered for treatment (Lambert et al., 2018; Goldmuntz, 2020; Legitimo et al., 2020).

Neuropsychiatric disorders (attention deficit hyperactivity disorder, autism spectrum disorder, schizophrenia, anxiety symptoms and sleep disturbances) are frequent in patients with 22q11.2 CNVs (Brzustowicz and Bassett, 2012; Moulding et al., 2020). Complex presentation of three or more psychiatric traits may occur in 73% of patients with 22q11.2 CNVs (Niarchou et al., 2014; Chawner et al., 2019; Niarchou et al., 2019). However, these were markedly underrepresented (19%) in our patient cohort (Table 4), and this is most probably due to the lack of awareness and screening rather than to their absence. This result further emphasizes the importance of multidisciplinary management of patients with 22q11.2 CNVs.

The large phenotypic variability of 22q11.2 microdeletions has recently been the focus of much research but is still not yet fully understood. The haploinsufficiency of the coding genes, including *TBX1, DGCR8, CRKL* among others, alone does not seem to account for the highly variable phenotypes and incomplete penetrance of affected individuals (Du et al., 2020). Some recent studies have investigated the role of possible genetic and epigenetic factors which contribute to the diversity of phenotypes associated with 22q11.2 deletions (Brzustowicz and Bassett, 2012; Bertini et al., 2017; Du et al., 2020). Breakpoint analysis of the LCR A-D region showed that small variations in the deletion size within this region have no significant role on phenotypic variability (Bertini et al., 2017). Pathogenic sequential variations in the remaining single copy of the genes (with an emphasis on *TBX1* gene) encompassed in the deleted region, were not yet revealed by previous investigation (Brzustowicz and Bassett, 2012). And this was further supported by our *TBX1* sequencing results, since no pathogenic *TBX1* variant was detected in our patients with 22q11.2 CNVs or in the overall CHD-cohort. Thus, pathogenic *TBX1* mutations may be causal most probably only in a small fraction of CHD patients and 22q11.2 CNV patients, if at all. It was also hypothesized that sequential variations elsewhere in the genome (for example, *de novo* mutations in histone modifying genes) may collectively contribute to this diversity (Zaidi et al., 2013). Bertini and colleagues have investigated additional rare and common CNVs in typical 22q11.2 patients (2017). According to their results, these additional CNVs often contain miRNA genes or mitochondrial genes, which may interact with 22q11.2 deletion and lead to metabolic and energetic problems rather than a decreased dosage of morphogenetic genes. The *DGCR8* gene is located within the typical 22q11.2 region and plays a crucial role in miRNA biosynthesis and, in combination with other CNV-miRNAs, may orchestrate highly variable phenotypic outcomes (Bertini et al., 2017). Previously, these additional CNVs, miRNAs have been investigated exclusively for 22q11.2 microdeletion patients, but not for duplication patients. Studying these duplication patients may further refine the diversity.

The family segregation study proved to be beneficial in cases with pathogenic CNVs, since in 18% further affected family members were identified. The number of familial cases was higher in our cohort than the previously described 6–10% (McDonald-McGinn et al., 1993-2020; Goldmuntz, 2020). The affected family members in our cohort exhibited similar or milder symptoms than the probands. This phenomenon has already been observed (Wozniak et al., 2010; Goldmuntz, 2020).

22q11.2 is considered one of the most unstable regions of the human genome, due to the low-copy repeat regions on chromosome 22. This instability predisposes the region to deletions and duplications through non-allelic homologous recombination events. Hence, the presence of a parental CNV may trigger the development of another CNV in the same or nearby chromosomal region in the offspring, as seen in the family P09 and as described by Capra et al. (2013).

In conclusion, based on the present results and on those described in the literature (Wozniak et al., 2010; Li et al., 2019; Goldmuntz, 2020), we suggest the implementation of the genetic screening of CNVs in the postnatal management of CHD patients, regardless of the type of CHDs. For this purpose, MLPA is a cost-effective, fast and specific method suitable for the screening of a large number of samples. Patients and families benefit greatly from early diagnosis, through the regular cardiological, orthopedic, endocrinological, immunological, neurodevelopmental, and psychiatric follow-ups, the more aggressive infection control and the possibility of positive family planning.

## Data Availability Statement

The original contributions presented in the study are included in the article/Supplementary Material, further inquiries can be directed to the corresponding author/s.

## Ethics Statement

The studies involving human participants were reviewed and approved by the National Medical Research Council (No. CHD-01/2016—IF-6299-8/2016) and by the Local Ethical Committee of the University of Szeged (No. 105/2016-SZTE). Written informed consent to participate in this study was provided by the participants’ legal guardian/next of kin.

## Author Contributions

DN and MS: conceptualization, review, editing, and supervision. DN, GZ, MO, AK, KH, GR, MK, AU and ON: methodology, investigation, and validation. DN and MO: data curation. GZ and DN: writing and original draft preparation. All authors contributed to the article and approved the submitted version.

## Conflict of Interest

The authors declare that the research was conducted in the absence of any commercial or financial relationships that could be construed as a potential conflict of interest.
