# Supplementary material for: Systemic Screening for 22q11.2 Copy Number Variations in Hungarian Pediatric and Adult Patients With Congenital Heart Diseases Identified Rare Pathogenic Patterns in the Region
Source: Front Genet. 2021 Apr 29;12:635480. doi: 10.3389/fgene.2021.635480 (PMC8117090; doi:10.3389/fgene.2021.635480)
Supplement: Supplementary file 1 [file Data_Sheet_1.docx]

**Supplementary Material**

1. **Supplementary Data**
   1. **Primers used for sequencing the coding regions of *TBX1* gene**

Primers were designed by using Primer3web version 4.1.0 (<http://primer3.ut.ee>) and NCBI Primer Blast (https://www.ncbi.nlm.nih.gov/tools/primer-blast/).

| **PRIMER** | **5’-3’** |
| --- | --- |
| TBX1 – 2 F | GTTCAGCATCGCCTCTCTG |
| TBX1 – 2 R | CTACCAAGAGCTGCCTCCAC |
| TBX1 – 3 F | ATCTCCGCCGTGTCCAG |
| TBX1 – 3 R | CGGCGGAGGATAGGTGTTAG |
| TBX1 – 4 F | CCCCAGGCAGGTCAAGG |
| TBX1 – 4 R | GACCCGCCACTTTCCAG |
| TBX1 – 5 F | AAGGCCCTCTGGGTTCAC |
| TBX1 – 5 R | ACAGGCCTCTTAGGGACAGG |
| TBX1 – 6 F | CTCCCACCCCAGATCCTC |
| TBX1 – 6 R | TTACACCCGCTTTTCCAGAG |
| TBX1 – 7/8 F | CTTGGTGCGCTTCTCCTAAC |
| TBX1 – 7/8 R | GAACCCGGATCCCACGAC |
| TBX1 – 9 F | ACTTGGGGTCTCGGGCAC |
| TBX1 – 9 R | GAACTTCGGGGCTGTGCAG |

- 1. **Primers and probes used in droplet digital PCR to determine the copy number changes in the controls**

***Primers and probe for TOP3B gene in droplet digital PCR***

NM_001282112.2: ***TOP3B*** – exon 7 (Chr22q11)

Forward primer: 5’ TCTCTCATCTCCTTTGGGCC 3’

Reverse primer: 5’ CTCTCAGAATCCATGCCCCA 3’

Probe: 5’ /HEX/ACCTTGGCCTGCAGCACCCA/BHQ/ 3’

***Reference primers and probe for PRDM15 gene in droplet digital PCR***

NM_001040424.3: ***PRDM15*** – intron 6-7 (Chr21)

Forward primer: 5’ ATGTTTCGCCAACTTCTGAG 3’

Reverse primer: 5’ AGAGCTATGGCACAAACCTG 3’

Probe: 5’ /FAM/AGGATTTGG/ZEN/GGCTGCGC 3’

1. **Supplementary Figures**
   1. **Positive MLPA results visualized with ratio charts by Coffalyser Software**


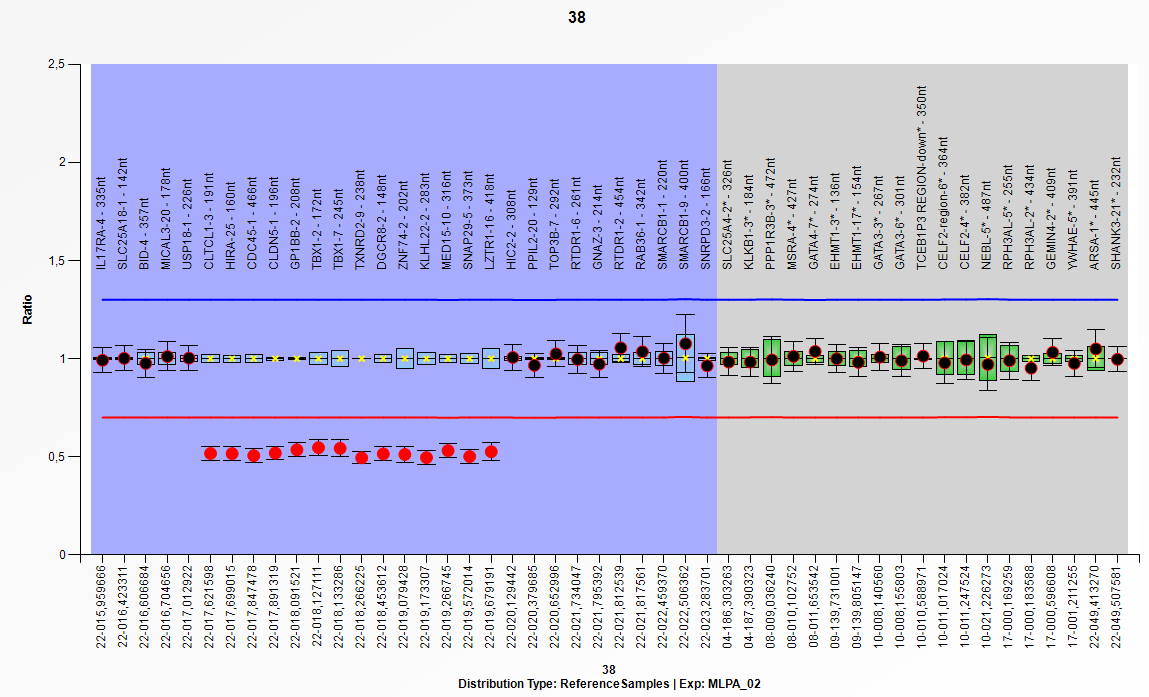
 **Classical LCR A-D deletion in probands P38, P104, P115, P138, P146, P208**


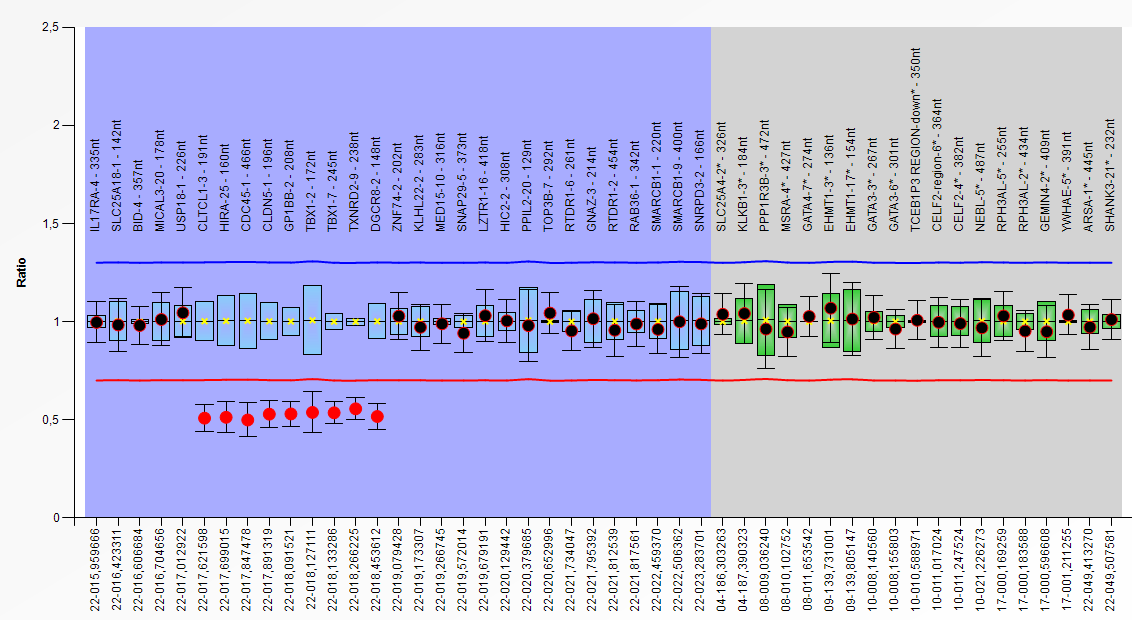
 **Nested proximal LCR A-B deletion in proband P39**


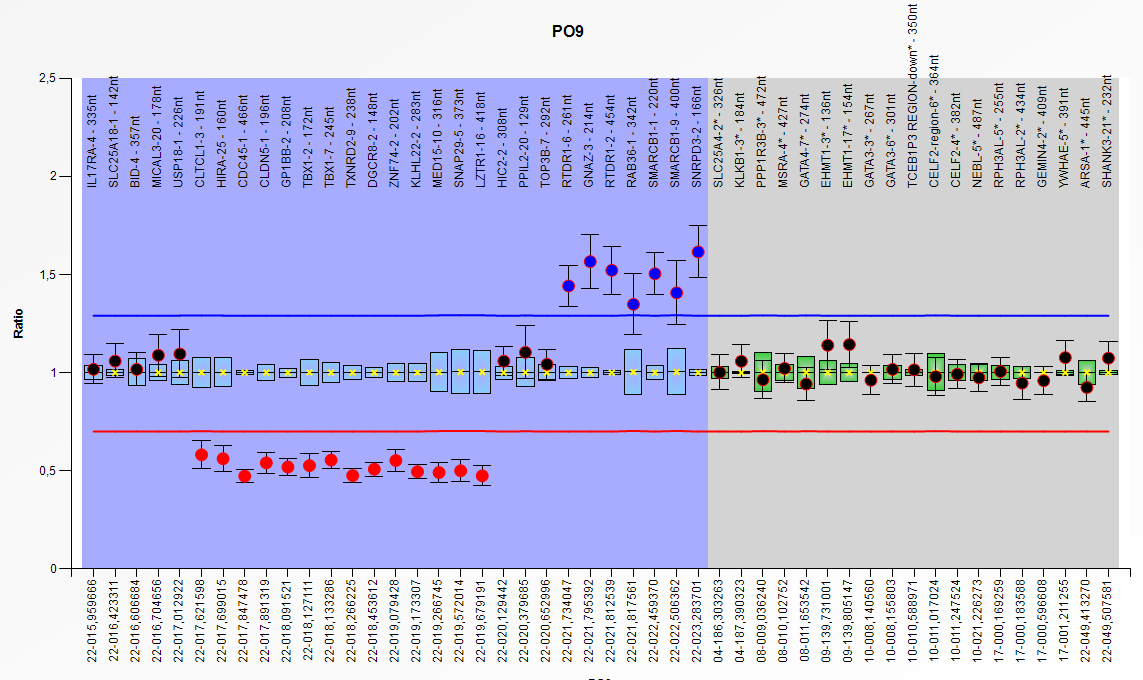
 **Classical LCR A-D deletion and distal LCR E-H duplication in proband P09 and her mother**


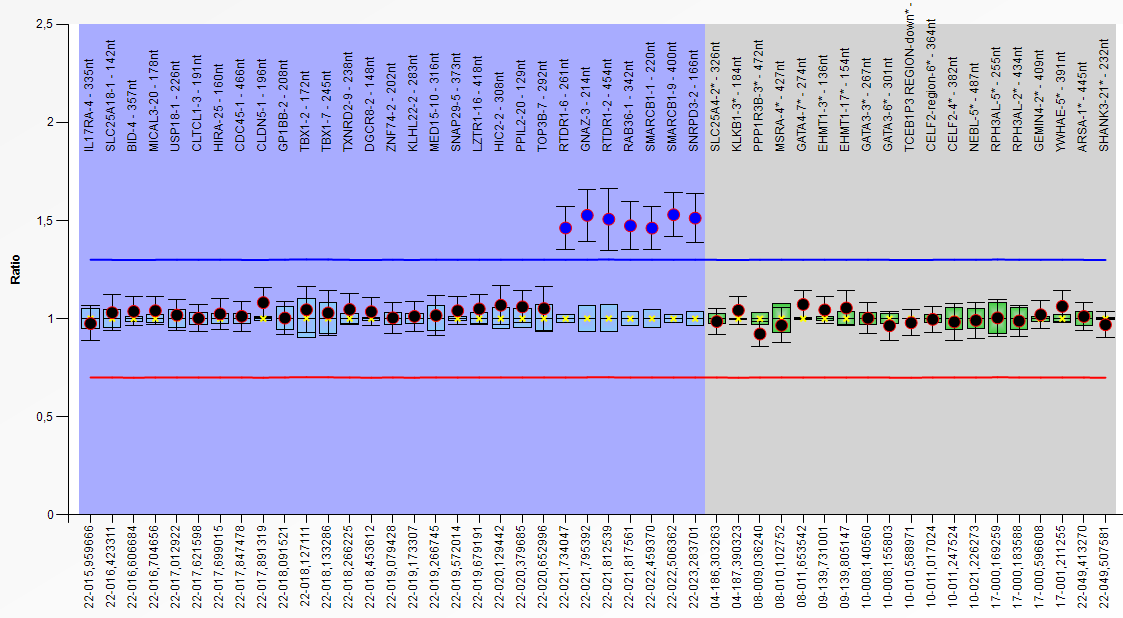


**Distal LCR E-H duplication in family members of proband P09**


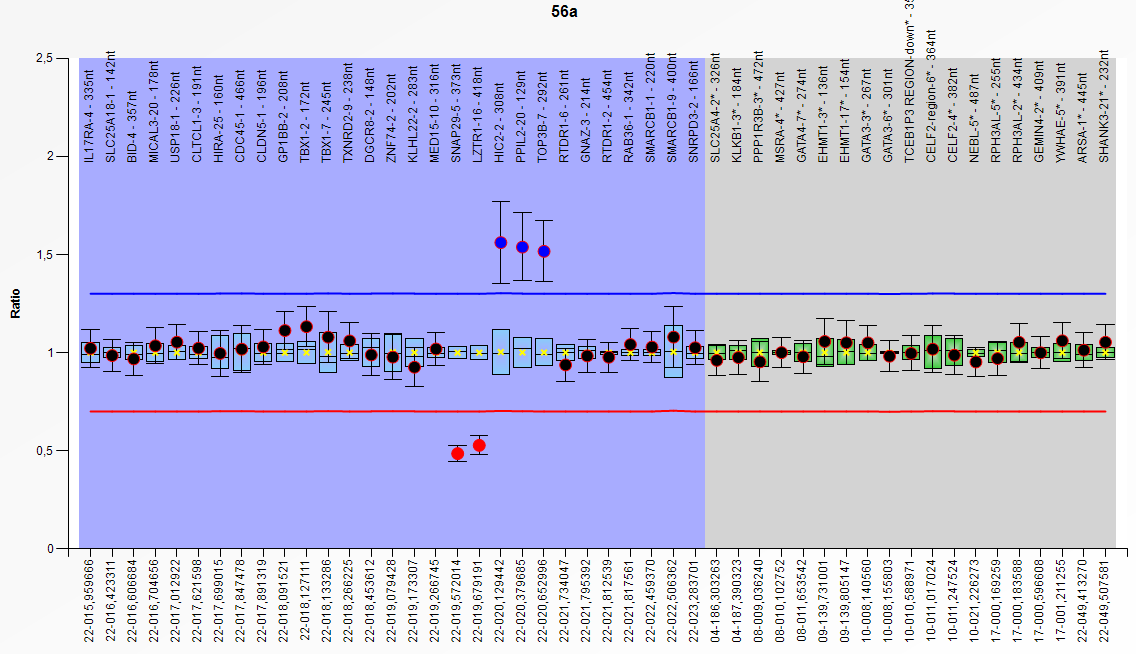


**Central LCR C-D deletion and LCR D-E duplication in proband P56 and in the proband’s mother**


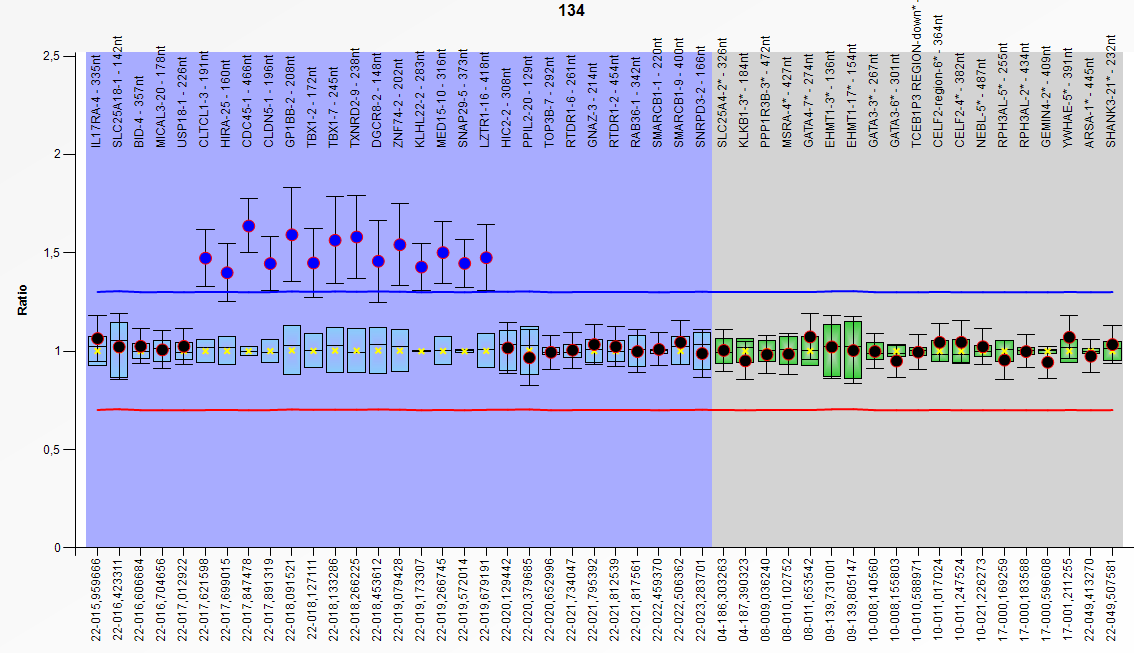


**Proximal LCR A-D duplication in probands P134 and P185**


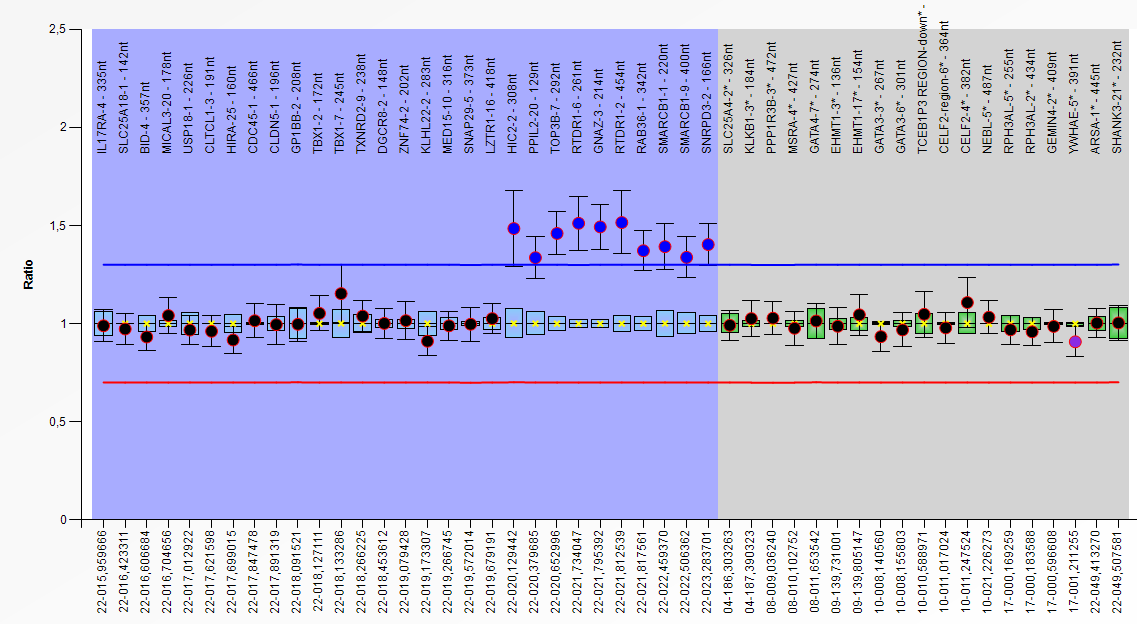


**Central-distal LCR D-H duplication in proband P209**


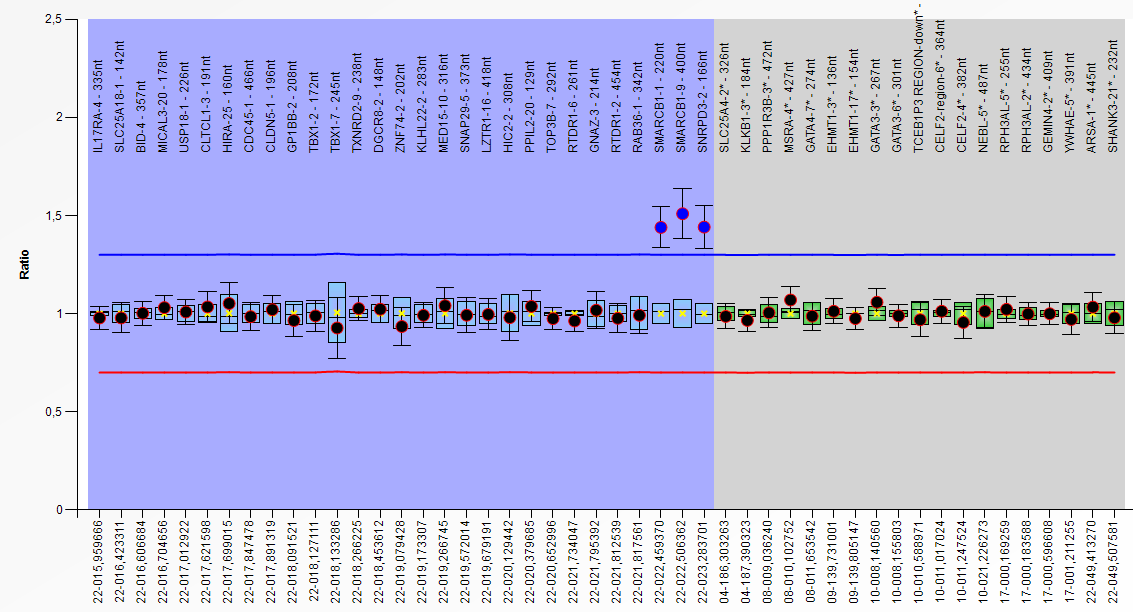


**Distal LCR F-H duplication in proband P165**


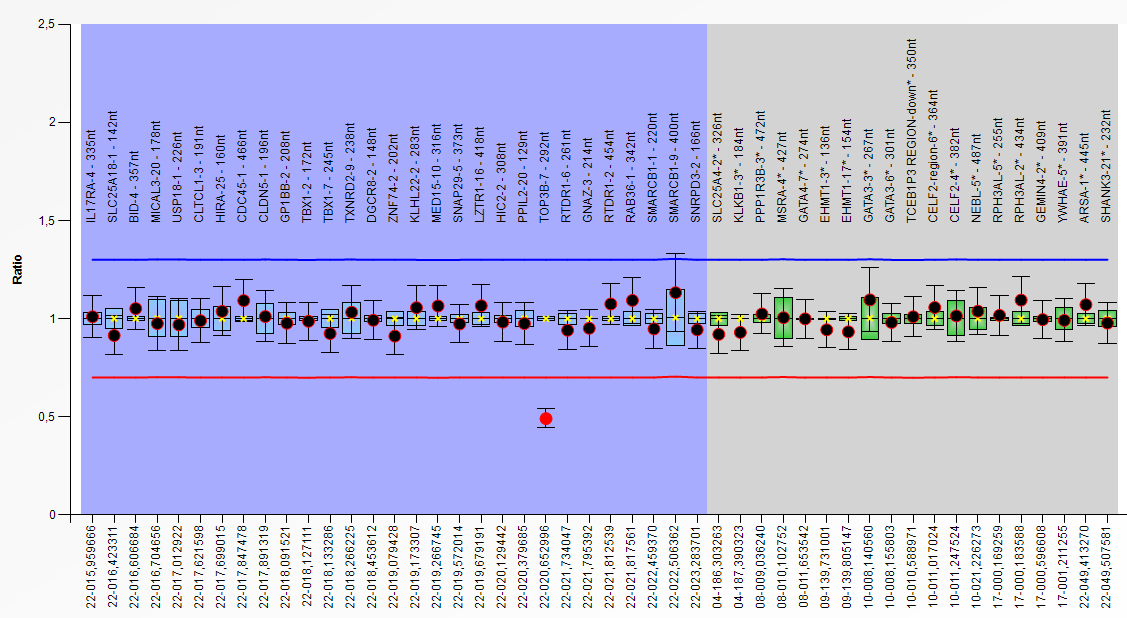


**TOP3B probe deletion in proband P54**


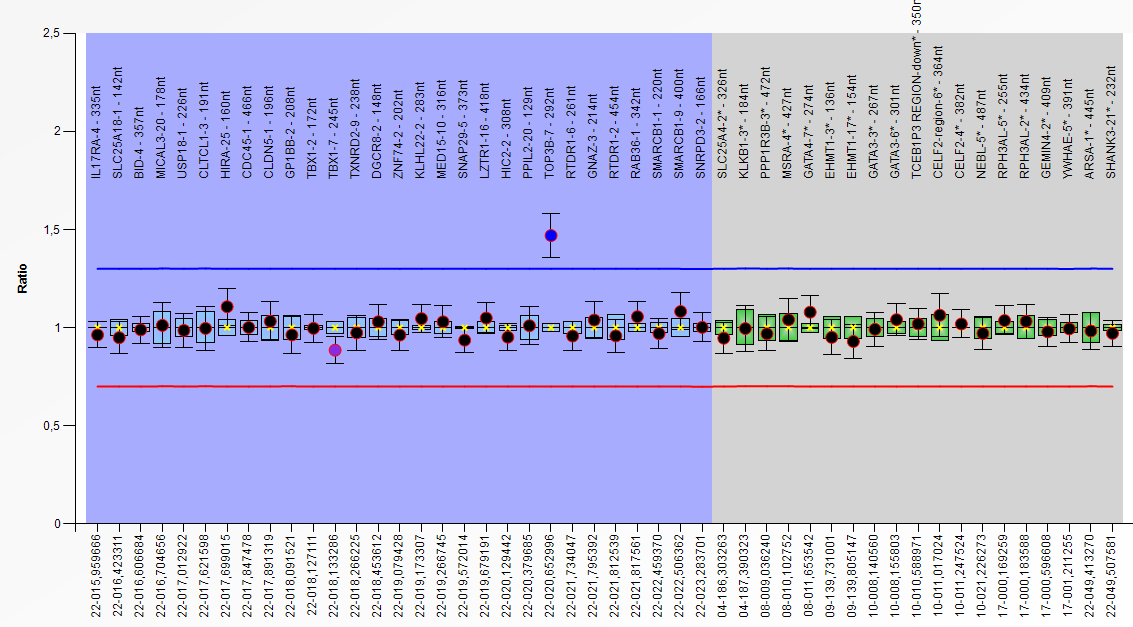


**TOP3B probe duplication in probands P51 and P69 and family members**


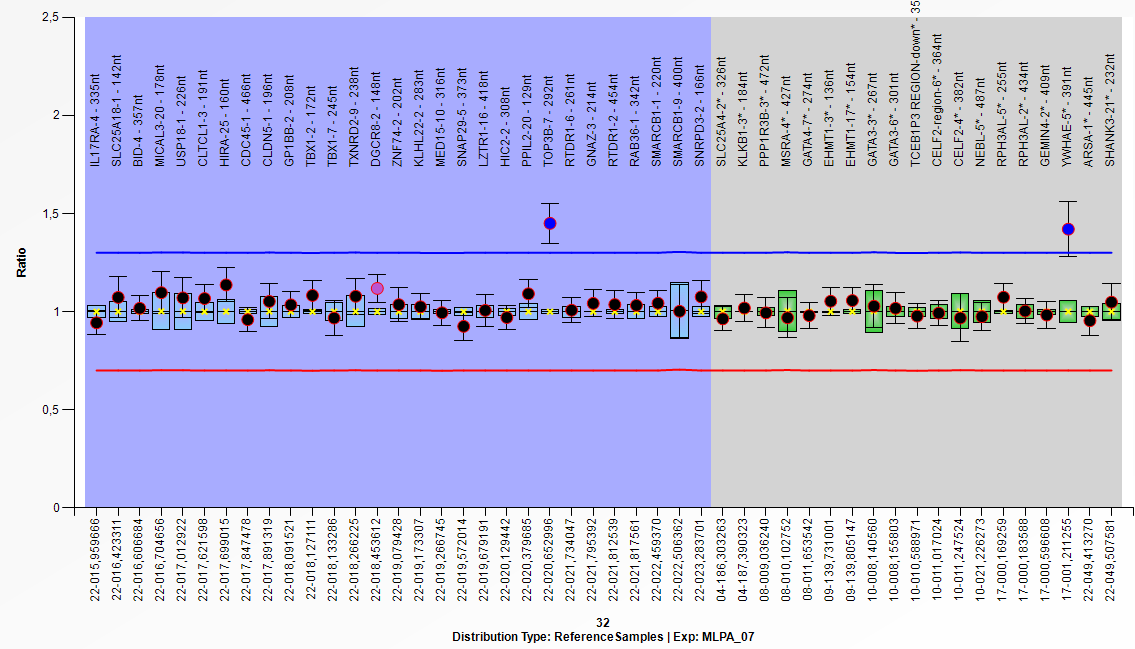


**TOP3B and YWHAE probe duplication in proband P32 and her mother**


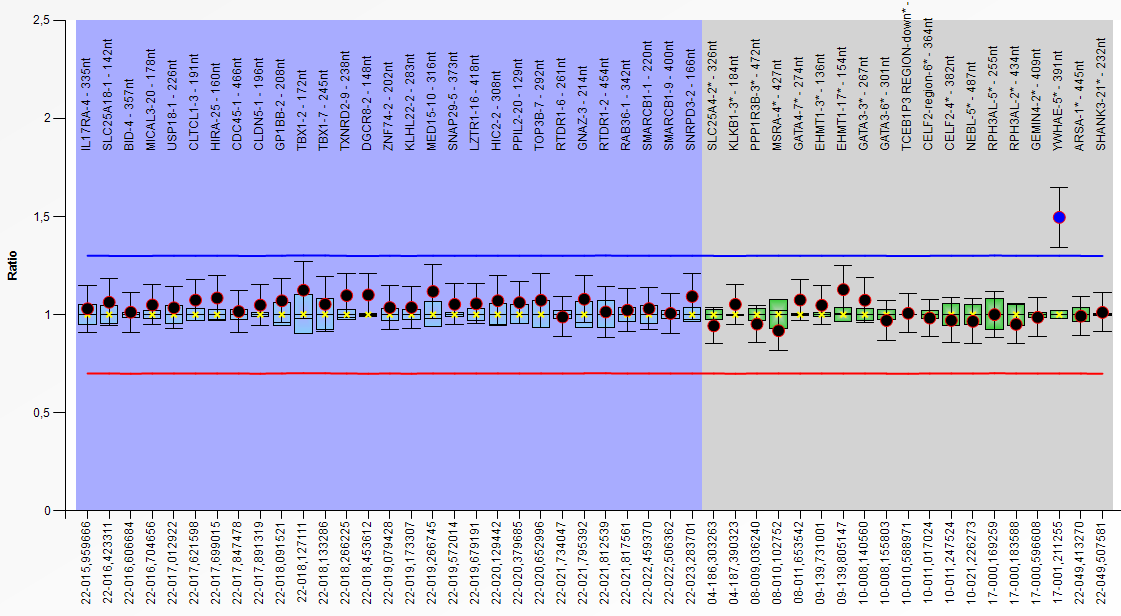
 **YWHAE probe duplication in family members of proband P32**

- 1. **Copy number variations confirmed by array-CGH (CytoScan 750K Array, Affymetrix)**


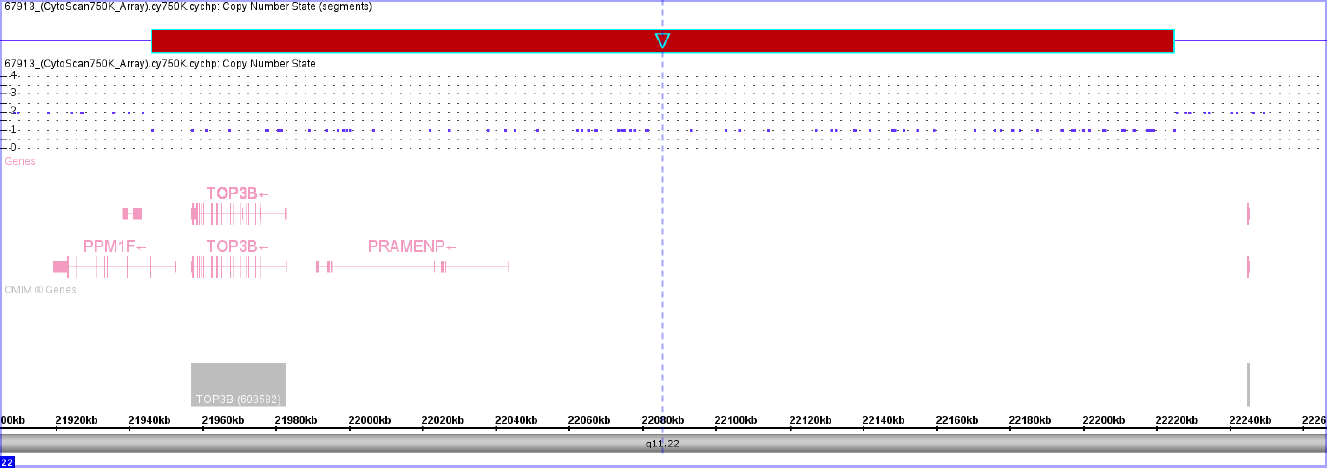


**278 kbp microdeletion in 22q11.22 in proband P54**

arr[GRCh38] 22q11.22(21,946,279-22,224,671)x1 *TOP3B* (#603582)

**
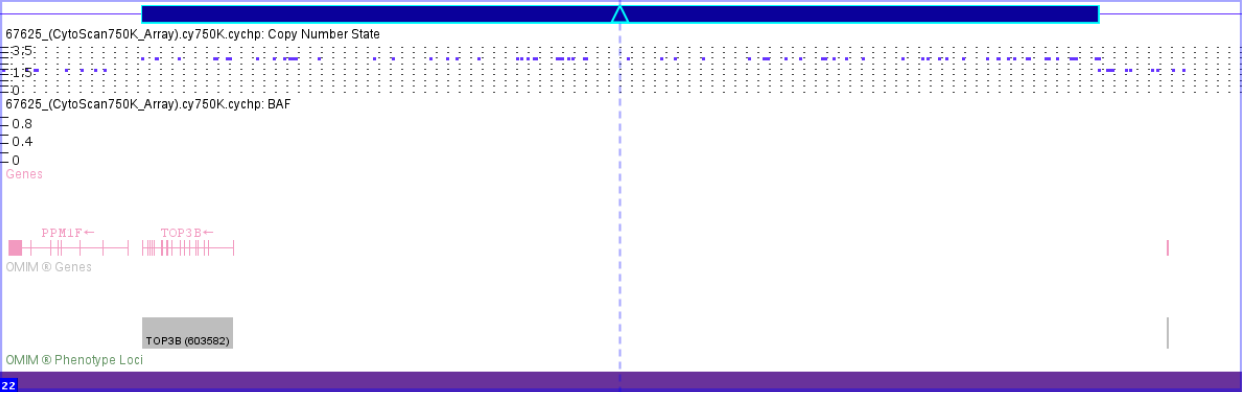
**

**268 kbp microduplication in 22q11.22 in proband P32**

arr[hg19] 22q11.22(22,311,348-22,579,775)x3 *TOP3B* (#603582)


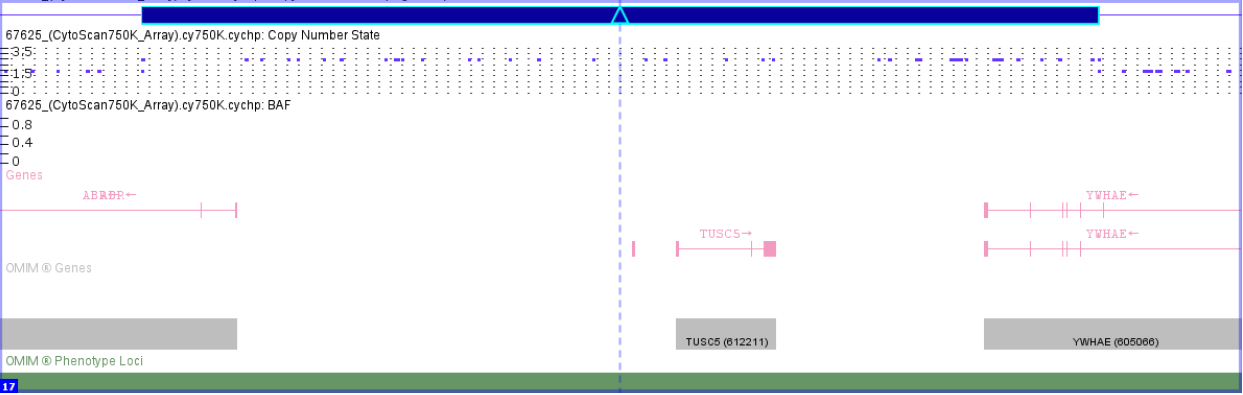


**201 kbp microduplication in 17p13.3 in proband P32**

arr[hg19] 17p13.3(1,070,538-1,271,913)x3

*ABR* (#600365), *TUSC5* (#612211), *YWHAE* (#605066)

- 1. **Droplet digital PCR results of *TOP3B* copy number variants in the patient and control samples**


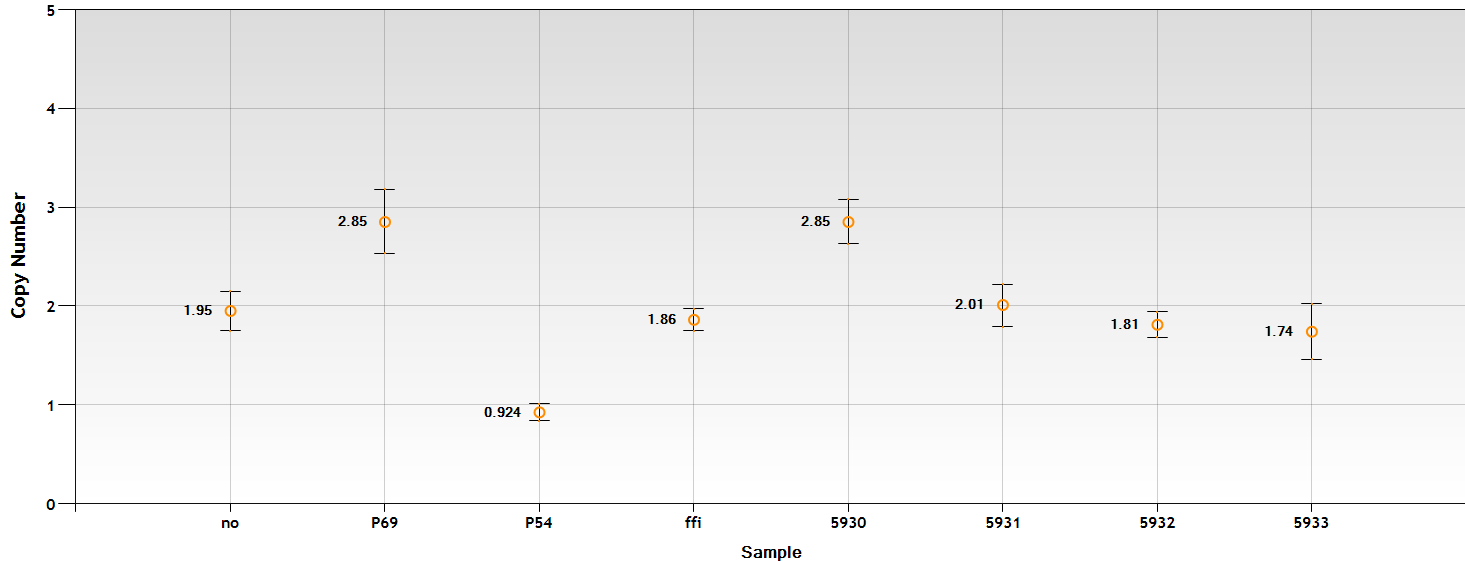


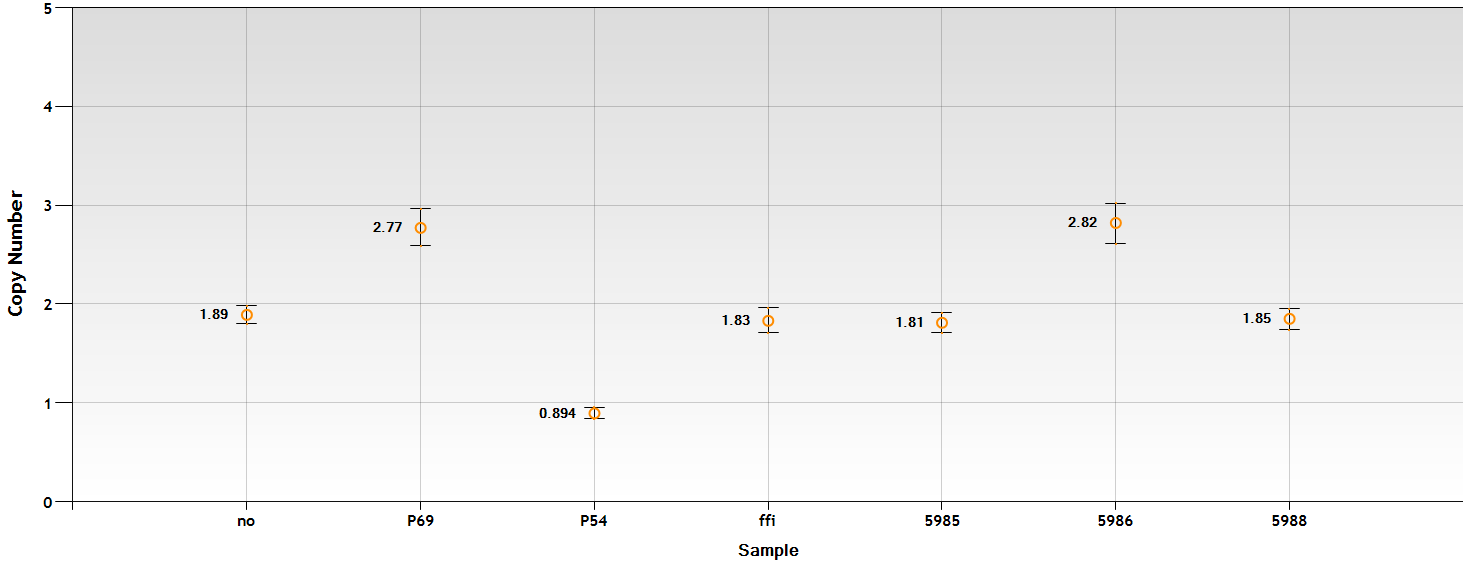


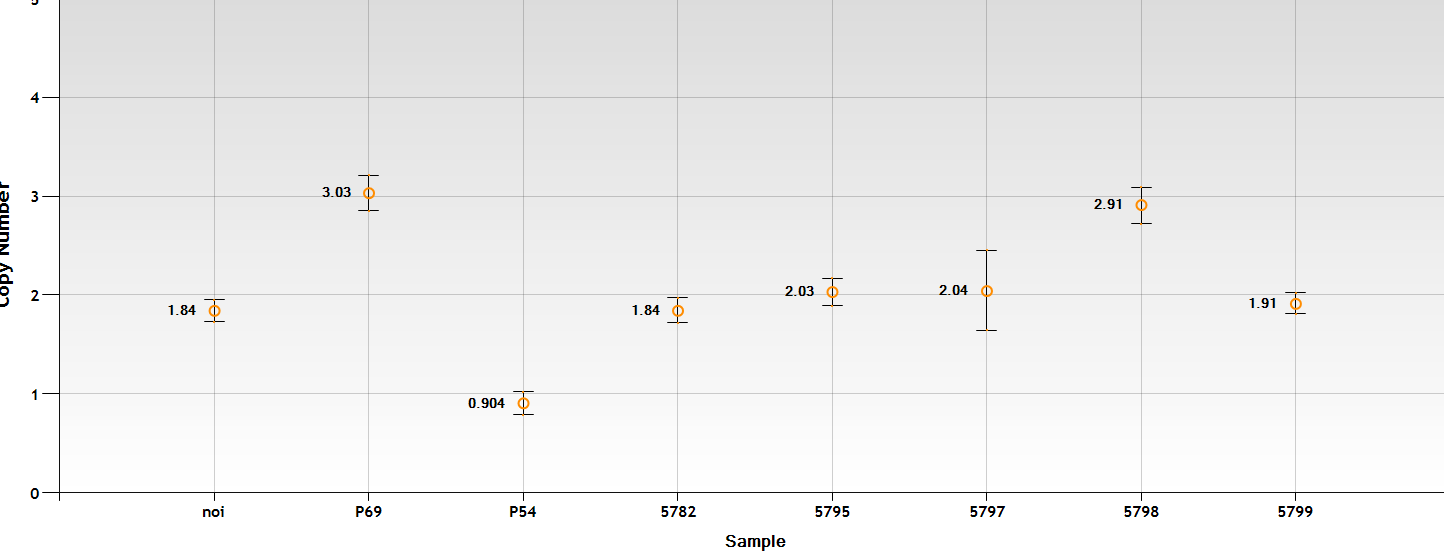


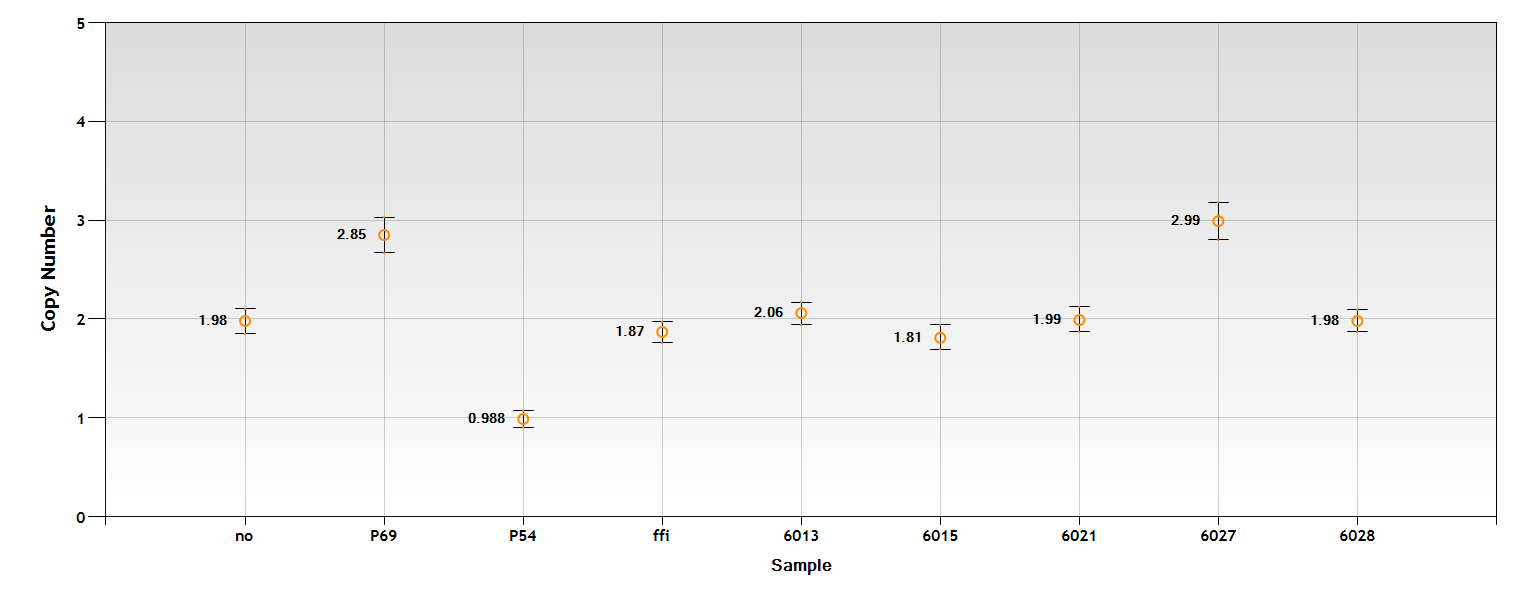


Reference samples with normal 2 copies of *TOP3B*: no, ffi. CHD-sample with *TOP3B* duplication: P69. CHD-sample with *TOP3B* deletion: P54. Healthy control samples with *TOP3B* duplication: 5930, 5986, 5798, 6027. All other samples are healthy controls with normal *TOP3B* copy number.
